# Diastolic dysfunction in individuals with and without heart failure with preserved ejection fraction

**DOI:** 10.1007/s00392-021-01907-x

**Published:** 2021-07-16

**Authors:** Jan-Per Wenzel, Ramona Bei der Kellen, Christina Magnussen, Stefan Blankenberg, Benedikt Schrage, Renate Schnabel, Julius Nikorowitsch

**Affiliations:** 1 Department of Cardiology, University Heart and Vascular Center Hamburg, Hamburg, Germany; 2 German Center for Cardiovascular Research (DZHK), Partner Site Hamburg/Kiel/Luebeck, Hamburg, Germany; 3 Epidemiological Study Center, Hamburg, Germany

**Keywords:** Diastolic dysfunction, ALVDD, HFpEF, Heart failure, Hamburg city health study, General population

## Abstract

**Aim:**

Left ventricular diastolic dysfunction (DD), a common finding in the general population, is considered to be associated with heart failure with preserved ejection faction (HFpEF). Here we evaluate the prevalence and correlates of DD in subjects with and without HFpEF in a middle-aged sample of the general population.

**Methods and results:**

From the first 10,000 participants of the population-based Hamburg City Health Study (HCHS), 5913 subjects (mean age 64.4 ± 8.3 years, 51.3% females), qualified for the current analysis. Diastolic dysfunction (DD) was identified in 753 (12.7%) participants. Of those, 11.2% showed DD without HFpEF (ALVDD) while 1.3% suffered from DD with HFpEF (DDwHFpEF). In multivariable regression analysis adjusted for major cardiovascular risk factors, ALVDD was associated with arterial hypertension (OR 2.0, *p *< 0.001) and HbA1c (OR 1.2, *p *= 0.007). Associations of both ALVDD and DDwHFpEF were: age (OR 1.7, *p *< 0.001; OR 2.7, *p *< 0.001), BMI (OR 1.2, *p *< 0.001; OR 1.6, *p *= 0.001), and left ventricular mass index (LVMI). In contrast, female sex (OR 2.5, *p *= 0.006), atrial fibrillation (OR 2.6, *p *= 0.024), CAD (OR 7.2, *p *< 0.001) COPD (OR 3.9, *p *< 0.001), and QRS duration (OR 1.4, *p *= 0.005) were strongly associated with DDwHFpEF but not with ALVDD.

**Conclusion:**

The prevalence of DD in a sample from the first 10,000 participants of the population-based HCHS was 12.7% of whom 1.3% suffered from HFpEF. DD with and without HFpEF showed significant associations with different major cardiovascular risk factors and comorbidities warranting further research for their possible role in the formation of both ALVDD and DDwHFpEF.

**Supplementary Information:**

The online version contains supplementary material available at 10.1007/s00392-021-01907-x.

## Introduction

Heart failure (HF) with preserved ejection fraction (HFpEF) is a widespread syndrome with increasing prevalence. It is characterized by clinical symptoms or signs, left ventricular ejection fraction ≥ 50%, and a pathological increase of cardiac filling pressures. HFpEF is associated with high morbidity and mortality, leading to medical and economic challenges [1]. Before the onset of HFpEF symptoms, such as dyspnoea on exertion, oedema, and fatigue, a process of structural and functional myocardial remodeling occurs [2]. Diastolic dysfunction (DD) plays a key role in the genesis of HFpEF [3]. It describes the successive disability of the left ventricle to properly relax during diastole, leading to an increase of left ventricular end-diastolic pressure. Asymptomatic left ventricular diastolic dysfunction (ALVDD) is an entity defined as the combination of diastolic abnormalities with normal left ventricular ejection fraction (LVEF) and the absence of symptoms. ALVDD revealed to be a significant predictor of fatal and non-fatal cardiovascular events and often progresses to symptomatic heart failure [4]. Reported prevalence of DD highly depends on the algorithm applied, ranging from as low as 1.3% according to the current recommended algorithms to 28% according to previous reports [5–7]. However, data on the current prevalence of DD and especially on ALVDD in the general population are scarce. The formation of ALVDD as well as its transition to HFpEF might be driven by risk factors and comorbidities such as age, diabetes, elevated blood pressure, and bodymass [8]. Nevertheless, factors associated both with ALVDD and DD with HFpEF are largely unknown. Furthermore, there is little evidence from population-based data on factors differentiating ALVDD from DD with HFpEF (DDwHFpEF).

Therefore, the present study investigated in a first step the prevalence of DD, ALVDD and HFpEF in the general population. Second, we evaluated risk factors associated with ALVDD and DD with HFpEF.

## Methods

### Study setting

**Fig. 1:**
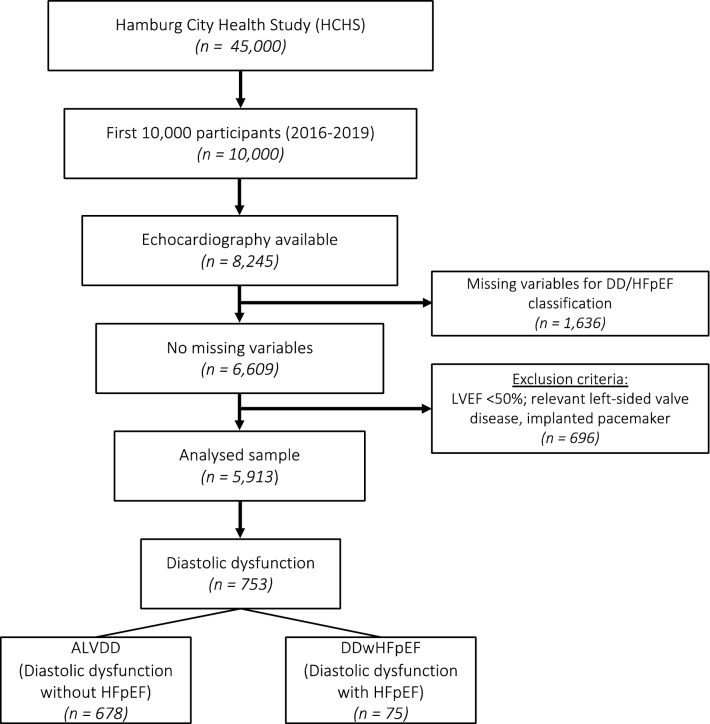
Study PRISMA. From a total of 8245 subjects providing a TTE examination, 1,636 subjects were excluded due to missing information for diastolic dysfunction and HFpEF classification. 696 met the exclusion criteria (pacemaker, AF, LVEF < 50% or relevant left-sided valve disease). Consequently, 5913 subjects were included in the study analysis, of whom 678 showed asymptomatic left ventricular diastolic dysfunction (ALVDD) and 75 diastolic dysfunction with heart failure with preserved ejection fraction (DDwHFpEF). *AF* atrial fibrillation, *DD* diastolic dysfunction, *LAVI* left atrial volume indexed to BSA, *LVEF* left ventricular ejection fraction

This study derived from the first 10,000 participants from the Hamburg City Health Study (HCHS, www.hchs.hamburg) recruited between 2016 and 2019. The HCHS is a single-centre, prospective, long-term, population-based cohort study placed in Hamburg, Germany [9]. 8,245 subjects of the first 10,000 participants received a transthoracic echocardiogram (TTE). Exclusion criteria were insufficient image quality to perform standardised measurements for grading DD, LVEF < 50%, atrial fibrillation (AF) at the day of the examination, moderate or severe mitral and aortic valve disease, lacking clinical or laboratory variables for HFpEF classification, and implanted pacemakers. Our final cohort comprised 5913 subjects (Fig. 1).

The research protocol of the study was approved by the HCHS steering board and the local ethics committee (PV5131, Medical Association Hamburg). All participants gave written informed consent.

### Anthropometric measurements, clinical, and laboratory data

All measurements were conducted by medical professionals at a baseline visit at the HCHS Epidemiological Study Centre in Hamburg following the published HCHS protocol [9]. Blood samples were withdrawn at the day of examination under fasting conditions. Demographics and clinical parameters were assessed by self-reported questionnaires and standardized interviews. Diabetes mellitus was determined by a fasting glucose level of ≥ 126 mg/dl, or the use of antidiabetic drugs. Coronary artery disease (CAD) was defined as suffering from one or more of the following conditions: status post myocardial infarction, percutaneous coronary intervention (PCI) or history of coronary bypass surgery assessed by questionnaire and personal medical record. Physical activity was defined as the number of hours per week spent on any type of sport. Socioeconomic-status-index (SES-index) was calculated by a combination of education, occupational status, and income. Quality of life was assessed by the validated SF-8™ (short form) health survey [10, 11].

### Echocardiographic data

Transthoracic echocardiography (TTE), carotid and abdominal aorta ultrasound examinations were performed and analysed by cardiologists and professional sonographers (technicians) at the baseline visit on dedicated ultrasound machines (Siemens Acuson SC2000 Prime, Siemens Healthineers, Erlangen, Germany) according to the guidelines of the American Society of Echocardiography (ASE) and the European Society of Cardiovascular Imaging (EACVI). All TTE standard views were assessed in two-dimensional echocardiography, including a three-dimensional four-chamber view for chamber quantification. Image analysis was performed using an off-line workstation with the commercially available Siemens syngo SC2000 version 4.0 software. Pulsed-wave Doppler examination of mitral inflow as well as Doppler tissue imaging of the mitral annulus were performed in each subject. Left ventricular ejection fraction as well as left-sided volumes were calculated using the two-dimensional biplane method of disks summation (modified Simpson’s rule).[6] Left ventricular mass (two-dimensional) was calculated according to the ASE and EACVI guidelines [6]. Tricuspid annular plane systolic excursion (TAPSE) was assessed by M-mode echocardiography in the apical four-chamber view. Right ventricular fractional area change (FAC) was measured in a right ventricular focused four-chamber view. Valvular heart disease was detected by a combination of colour Doppler and continuous wave-Doppler following the current ASE and EACVI guidelines [12].

### Classification of diastolic function and HFpEF

Diastolic function was assessed on the basis of three conditions: average E/eʹ ratio > 10; septal eʹ < 7 cm/s or lateral eʹ < 10 cm/s, and left atrial volume index (LAVI) > 34 ml/m^2^. DD was considered to be present if ≥ 2 conditions were positive.

For the diagnosis of HFpEF, the 2016 ESC guidelines for the diagnosis and treatment of acute and chronic heart failure were applied [13]. HF was considered present if subjects showed the combination of symptoms/signs, laboratory data, and echocardiographic criteria. Symptoms and signs included: oedema, dyspnoea, history of heart failure or medication (loop diuretics, aldosterone antagonists). Subjects presenting with preserved LVEF (LVEF ≥ 50%), NT-proBNP levels exceeding 125 pg/ml, symptoms or signs of HF and either left ventricular hypertrophy (defined as LV mass indexed to BSA > 95 g/m^2^ for women, > 115 g/m^2^ for men), left atrial enlargement (defined as LAVI > 34 ml/m^2^) or DD were classified as HFpEF individuals. In our final analysis, we only included those subjects with HFpEF who were diagnosed with DD.

### Peripheral arteries and abdominal aorta

Carotid and femoral arteries were assessed using a linear array transducer (Siemens Acuson S2000 9L4, 4–9 MHz) at high-resolution B-mode. Images of the common carotid artery (CCA) were obtained, and intima-media thickness (IMT) was measured 10 mm caudal of the carotid bulb at three different points. IMT was assessed for the both sides and averaged. Pulsed-wave-Doppler-mode was used to evaluate the flow-velocity of the internal carotid artery (ICA) and the common femoral artery (CFA). The ankle-brachial index was measured using a sphygmomanometer and Doppler probe. The score for both sides was calculated, and the lower one was chosen. The abdominal aorta was examined using a convex transducer (Siemens Acuson S2000 6C1 HD, 1.5–6 MHz). It was assessed in strict orthogonal orientation until the aortic bifurcation, and the largest outer-to-outer-wall diameter was assessed manually. Pulsed-wave-Doppler-mode was used to measure the peak systolic velocity.

### Statistical analysis

Given the large sample size, normality of continuous variables was assessed utilising normal Q–Q plots. Magnitudes of continuous variables were presented as mean ± standard deviation (SD) or median ± interquartile range (IQR), accordingly. Intra-class correlation coefficient (ICC) estimates and their 95% confident intervals (CI) were calculated based on a mean-rating, consistency, two-way mixed-effects model [14].

The unpaired *t* test was used to analyse differences between groups. For non-normally distributed variables, the Mann–Whitney *U* test was used instead. Pearson’s correlation coefficient was used to quantify the correlation between end-diastolic and mid-systolic measurements. For multiple group comparisons, overall significance levels were obtained using one-way ANOVA. For multiple pairwise comparison against the base-mean, the *t* test was used.

Multivariable logistic regression was used to assess the association between ALVDD and DDwHFpEF with multiple possible risk factors, biomarkers, and echocardiographic variables. To determine the influence of different risk factor profiles on ALVDD and DDwHFpEF, a binomial logistic regression model was calculated with ALVDD or DDwHFpEF as the dependent variable and the respective variables of the profiles as predictors (age, sex, diabetes, hypertension, BMI, GFR, CAD, AF). The numerical variables (age, BMI, GFR) were categorized accordingly beforehand. Based on these models, the probabilities for ALVDD and DDwHFpEF of the profiles were estimated. Differences were considered statistically significant at a two-sided *p* value level of 0.05. All statistical analyses were performed using R (version 3.5.1). A list of the used packages and versions can be found in the appendix.

## Results

### Baseline characteristics

The analysed sample of 5,913 participants from the first 10,000 HCHS participants showed the characteristics of a representative middle-aged (mean age 64.4 ± 8.3 years) European population with 3032 women (51.3%) (Table 1).

**Table 1:** Baseline characteristics of the study population

	Normal diastolic function	ALVDD (DD without HFpEF)	DDwHFpEF (DD with HFpEF)	*p* value
*n* (%)	5160 (87.3)	678 (11.5)	75 (1.3)	
Demographics				
Age, years	60.0 [54.0, 67.0]	67.0 [61.0, 72.0]	71.0 [66.5, 73.0]	< 0.001
Female	2642 (51.2)	344 (50.7)	46 (61.3)	0.209
Ethnicity				0.173
White	5045 (97.8)	662 (97.6)	74 (98.7)	
Black	20 (0.4)	4 (0.6)	1 (1.3)	
Asian	33 (0.6)	9 (1.3)	0 (0.0)	
Other	62 (1.2)	3 (0.5)	0 (0.0)	
BMI, kg/m^2^	25.7 [23.3, 28.6]	26.8 [24.3, 29.7]	27.9 [25.2, 31.9]	< 0.001
Current skmoker	1055 (20.6)	140 (20.8)	19 (25.3)	0.596
Education short**	2602 (52.9)	378 (59.1)	50 (71.4)	< 0.001
Physical scales***	53.1 [47.6, 56.7]	52.6 [46.6, 56.3]	43.1 [35.3, 51.6]	< 0.001
Mental scales***	57.2 [51.1, 58.5]	57.4 [52.6, 58.8]	56.5 [48.5, 57.8]	0.011
Quality of life***	0.9 [0.9, 1.0]	0.9 [0.9, 1.0]	0.9 [0.8, 0.9]	< 0.001
Comorbidities				
Hypertension	2882 (59.1)	529 (79.9)	69 (93.2)	< 0.001
Diabetes	300 (6.3)	72 (11.2)	20 (28.6)	< 0.001
Allergies	1885 (41.9)	231 (39.7)	28 (45.2)	0.511
Coronary artery disease	208 (5.5)	51 (10.2)	24 (47.1)	< 0.001
Atrial fibrillation	144 (2.8)	33 (4.9)	12 (16.9)	< 0.001
COPD	297 (6.3)	38 (6.1)	20 (31.7)	< 0.001
OSAS	229 (4.8)	47 (7.5)	13 (19.1)	< 0.001
Peripheral artery disease	118 (2.5)	24 (3.9)	6 ( 8.8)	0.001
Chronic venous insufficiency	134 (4.6)	27 (6.9)	4 (11.1)	0.038
Dyspnoea	370 (7.9)	42 (6.7)	56 (78.9)	< 0.001
Oedema	28 (1.0)	3 (0.8)	4 (11.1)	< 0.001
Biological data + medication				
Timed up and go Time, s	7.0 [6.0, 8.0]	7.0 [6.0, 8.0]	8.0 [7.0, 10.0]	< 0.001
Ankle-branchial index	1.1 [1.0, 1.2]	1.0 [1.0, 1.1]	1.1 [1.0, 1.1]	0.021
Intima-media-thickness	0.7 [0.7, 0.8]	0.8 [0.7, 0.9]	0.8 [0.8, 0.9]	< 0.001
ICA peak systolic velocity, m/s	96.2 [81.6, 113.8]	95.3 [82.9, 111.3]	98.3 [84.7, 112.1]	0.704
Abdominal aorta diameter, mm	17.7 [16.1, 19.5]	18.1 [16.0, 20.1]	17.4 [15.9, 19.5]	0.018
Aldosterone antagonists	18 (0.4)	3 (0.5)	3 (4.2)	< 0.001
Loop diuretics	55 (1.1)	11 (1.7)	8 (11.3)	< 0.001
Betablocker	591 (12.0)	136 (20.7)	39 (54.9)	< 0.001
ACEi/ARBs	868 (17.7)	180 (27.4)	32 (45.1)	< 0.001
Laboratories				
LDL, mg/dl	122.0 [98.0, 146.0]	121.0 [94.0, 146.0]	103.0 [81.0, 138.0]	0.022
GFR, ml/min	85.7 [75.5, 95.0]	85.0 [74.0, 94.0]	74.7 [62.8, 84.8]	< 0.001
NT-proBNP, ng/l	68.0 [39.0, 114.0]	78.0 [50.0, 116.5]	233.0 [175.5, 379.5]	< 0.001
hsCRP, mg/dl	0.1 [0.1, 0.2]	0.1 [0.1, 0.3]	0.2 [0.1, 0.5]	< 0.001
TSH, U/l	1.2 [0.8, 1.7]	1.1 [0.8, 1.6]	1.1 [0.7, 1.6]	0.296
Hemoglobin, g/dl	14.3 [13.6, 15.1]	14.4 [13.7, 15.1]	13.9 [13.2, 14.6]	0.004
HbA1c, %	5.5 [5.3, 5.7]	5.6 [5.4, 5.9]	5.7 [5.4, 6.3]	< 0.001

Continuous variables are presented as mean ± standard deviation or median ± interquartile range. Categorical variables are presented as absolute numbers and percentages. *p* value for intergroup differences

*ACEi* angiotensin-converting enzyme inhibitor, *ARB* angiotensin receptor blocker, *BMI* body mass index, *COPD* chronic obstructive pulmonary disease, *GFR* glomerular filtration rate, *LDL* low-density lipoprotein, *NT-proBNP* N-terminal pro-B-type natriuretic peptide, *OSAS* obstructive sleep apnoea syndrome, *TSH* thyroid-stimulating hormone

** ISCED (International Standard classification of education) classification 1 and 2

*** Scores of the EQ-5D (European Quality of Life 5 Dimensions 3 Level Version) and SF-8 (Short Form 8 Health Survey)

DD was diagnosed in 753 subjects (12.7%). Of those, 75 (1.3%) subjects suffered from HFpEF (DDwHFpEF) and 678 (11.5%) participants were asymptomatic (ALVDD). Individuals with DDwHFpEF were older, and in contrast to individuals with ALVDD and normal diastolic function predominantly female. The prevalence of most cardiovascular risk factors gradually increased from normal diastolic function to ALVDD to DDwHFpEF, including arterial hypertension, diabetes, current smoking, coronary artery disease (CAD), and atrial fibrillation (AF). The use of heart failure medication as well as the levels of the biomarkers NT-proBNP, hsCRP and HbA1c accordingly increased between the three groups. However, the glomerular filtration rate (GFR), low-density lipoprotein (LDL), and haemoglobin showed an inverse pattern (Table 2). Electrocardiographically, the PR interval, the duration of the QRS complex and the corrected QT interval significantly increased from normal to DDwHFpEF. LVEF was similar in all groups. Nevertheless, there was a significant increase for LV mass index, LVEDV as well as markers of diastolic dysfunction such as E/e’ ratio, TR Vmax, and LASV between the three groups (Table 2).

**Table 2:** Echocardiographic and electrocardiographic characterisation of the study population

	Normal diastolic function	ALVDD (DD without HFpEF)	DDwHFpEF (DD with HFpEF)	*p* value
*n* (%)	5160 (87.3)	678 (11.5)	75 (1.3)	
ECG data				
PR, ms	160.0 [146.0, 178.0]	166.0 [153.0, 182.0]	176.0 [150.0, 192.0]	< 0.001
QRS, ms	92.0 [86.0, 98.0]	92.0 [86.0, 100.0]	94.0 [88.0, 104.0]	0.005
QTc, ms	418.0 [405.0, 432.0]	423.0 [408.0, 438.0]	431.0 [417.0, 445.0]	< 0.001
Hemiblock	171 (3.7)	23 (3.8)	8 (11.6)	0.003
AV-block	233 (5.0)	50 (8.3)	13 (18.8)	< 0.001
Echocardiographic data				
LVEF, %	59.0 [56.4, 62.2]	58.4 [56.0, 61.4]	58.5 [55.8, 61.0]	0.001
LV mass index, g/m^2^	79.9 [69.8, 92.5]	87.9 [76.2, 104.4]	94.6 [80.8, 115.6]	< 0.001
LVEDV, ml	108.4 [90.8, 130.1]	116.3 [94.4, 137.3]	109.2 [88.4, 143.0]	< 0.001
LV lateral eʹ, cm/s	10.8 [9.0, 12.8]	7.9 [6.6, 9.0]	7.7 [6.4, 9.5]	< 0.001
LV septal eʹ, cm/s	8.8 [7.5, 10.4]	6.7 [5.8, 7.8]	6.2 [5.5, 7.2]	< 0.001
E/eʹ mean ratio	7.0 [6.0, 8.1]	10.4 [8.6, 11.3]	11.1 [9.3, 12.4]	< 0.001
E/A ratio	1.0 [0.8, 1.2]	0.9 [0.7, 1.0]	0.8 [0.7, 1.0]	< 0.001
TR Vmax, m/s	2.1 [1.9, 2.3]	2.2 [1.9, 2.4]	2.3 [2.0, 2.5]	< 0.001
TAPSE, mm	2.4 [2.2, 2.7]	2.4 [2.2, 2.7]	2.3 [1.9, 2.6]	0.066
RV sʹ, cm/s	0.1 [0.1, 0.2]	0.1 [0.1, 0.2]	0.1 [0.1, 0.2]	0.101
LASV, ml	25.2 [21.2, 29.5]	34.1 [24.5, 37.8]	35.1 [27.9, 40.1]	< 0.001

Continuous variables are presented as median ± interquartile range. Categorical variables are presented as absolute numbers and percentages. *p* value for intergroup differences

*LASV* left atrial systolic volume, *LVEDD* left ventricular end-diastolic diameter, *LV* left ventricle, *LVEDV* left ventricular end-diastolic volume, *LVEF* left ventricular ejection fraction, *LVMI* left ventricular mass index, *RV* right ventricle, *TAPSE* tricuspid annular peak systolic excursion

### Correlates of ALVDD and DDwHFpEF

**Fig. 2:**
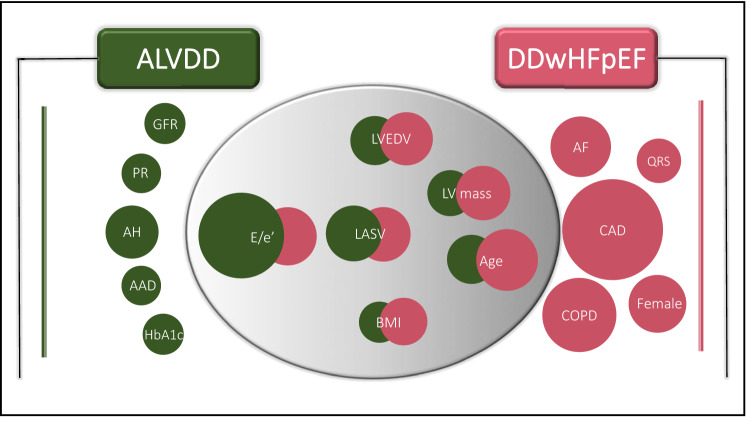
Pertinent associated factors for ALVDD and diastolic dysfunction with HFpEF. Figure 2 visualizes ORs derived from adjusted logistic regression analysis*.* The size of the circles correlates with the size of the OR. Adjustment was performed for age, gender, BMI, hypertension, diabetes, coronary artery disease (CAD), and smoking. All displayed ORs showed statistical significance. *AAD* Abdominal aorta diameter, *AF* atrial fibrillation, *AH* arterial hypertension, *ALVDD* asymptomatic left ventricular diastolic dysfunction, *BMI* body mass index, *CAD* coronary artery disease, *GFR* glomerular filtration rate, *DDwHFpEF* diastolic dysfunction with heart failure with preserved ejection fraction, *hsCRP* high sensitivity C-reactive protein, *LASV* left atrial end-systolic volume, *LVEDV* left ventricular end-diastolic volume, *LV mass* left ventricular mass, *QRS* QRS duration, *QoL* quality of life, *PR* PR interval

Several demographic, functional, and morphological data, as well as biomarkers were associated with ALVDD, DDwHFpEF or both cohorts (Table 3, Fig. 2) after adjustment for age, sex, BMI, hypertension, diabetes, current smoking, and CAD*.* ALVDD correlated with arterial hypertension with an OR of 2.0 (95% CI 1.5–2.6, *p *< 0.001), HbA1c with an OR of 1.2 (95% CI 1.1–1.3, *p *= 0.007), renal function assessed by glomerular filtration rate with an OR of 1.2 (95% CI 1.1–1.4, *p *< 0.001), the PR interval with an OR of 1.1 (95% CI 1.0–1.3) and the diameter of the abdominal aorta with an OR of 1.1 (95% CI 1.0–1.2, *p *= 0.01). Cardiovascular risk factors that were not only associated with ALVDD but also with DDwHFpEF included BMI (OR 1.2, 95% CI 1.1–1.3, *p *< 0.001; OR 1.6, 95% CI 1.2–2.1, *p *= 0.001) and age (OR 1.7, 95% CI 1.5–1.9, *p *< 0.001; OR 2.7, 95% CI 1.8–4.3, *p *< 0.001). Furthermore, the functional and morphological echocardiographic parameters E/e’, left ventricular end-diastolic volume (LVEDV), left atrial systolic volume (LASV), and left ventricular mass indexed to BSA showed significant associations with both ALVDD and DDwHFpEF. Apart from E/eʹ, the associations were generally stronger for DDwHFpEF than for ALVDD.

**Table 3:** Multivariable logistic regression analysis for the association of ALVDD and DDwHFpEF with specific risk factors

	ALVDD (DD without HFpEF)		DDwHFpEF (DD with HFpEF)	
	OR (95% CI)	*p* value	OR (95% CI)	*p* value
Demographics				
Age	1.7 (1.5–1.9)	< 0.001	2.7 (1.8–4.3)	< 0.001
Female	1.2 (1.0–1.4)	0.141	2.5 (1.3–4.9)	0.006
BMI	1.2 (1.1–1.3)	< 0.001	1.6 (1.2–2.1)	0.001
Current skmoker	1.3 (1.0–1.7)	0.055	2.1 (1.0–4.4)	0.051
Education short**	1.1 (0.9–1.3)	0.506	1.2 (0.6–2.4)	0.646
Physical scales***	1.0 (0.9–1.2)	0.611	0.7 (0.5–0.9)	0.002
Mental scales***	1.0 (0.9–1.1)	0.971	0.8 (0.6–1.1)	0.1
Quality of life***	1.0 (0.9–1.1)	0.993	0.8 (0.7–1.0)	0.063
Comorbidities				
Hypertension	2.0 (1.5–2.6)	< 0.001	2.8 (0.9–12.0)	0.101
Diabetes	1.2 (0.8–1.6)	0.383	1.7 (0.8–3.6)	0.165
Allergies	1.0 (0.8–1.3)	0.807	1.2 (0.6–2.3)	0.69
Coronary artery disease	1.1 (0.8–1.5)	0.595	7.2 (3.6–14.2)	< 0.001
Atrial fibrillation	0.9 (0.6–1.5)	0.769	2.6 (1.1–5.9)	0.024
COPD	0.6 (0.4–1.0)	0.049	3.9 (1.8–8.0)	< 0.001
OSAS	1.3 (0.8–1.9)	0.275	1.5 (0.5–3.8)	0.395
Peripheral artery disease	1.5 (0.9–2.5)	0.111	0.5 (0.1–1.9)	0.396
Chronic venous insufficiency	0.8 (0.4–1.3)	0.342	0.9 (0.1–3.3)	0.867
Biological data + medication				
Timed up and go Time	0.9 (0.8–1.1)	0.397	1.3 (0.9–1.7)	0.144
Ankle-branchial index	0.9 (0.8–1.1)	0.348	1.2 (0.8–2.1)	0.419
Intima-media-thickness	1.0 (0.9–1.2)	0.517	1.2 (0.9–1.5)	0.24
ICA peak systolic velocity	1.0 (0.9–1.1)	0.936	1.0 (0.7–1.4)	0.985
Abdominal aorta diameter	1.1 (1.0–1.2)	0.01	0.9 (0.7–1.2)	0.534
Laboratories				
LDL	1.0 (0.9–1.1)	0.574	0.9 (0.7–1.3)	0.73
GFR	1.2 (1.1–1.4)	< 0.001	1.0 (0.7–1.3)	0.781
hsCRP	1.0 (0.9–1.1)	0.932	1.2 (0.9–1.3)	0.079
TSH	1.0 (0.9–1.1)	0.843	1.0 (0.7–1.2)	0.932
Hemoglobin	1.0 (0.9–1.1)	0.827	0.9 (0.6–1.2)	0.415
HbA1	1.2 (1.0–1.3)	0.007	0.9 (0.7–1.3)	0.759
ECG data				
PR	1.1 (1.0–1.3)	0.013	1.0 (0.7–1.2)	0.75
QRS	1.0 (0.9–1.1)	0.459	1.4 (1.1–1.7)	0.005
QTc	1.0 (0.8–1.1)	0.847	1.0 (0.7–1.1)	0.758
Hemiblock	0.6 (0.4–1.1)	0.109	1.4 (0.4–3.8)	0.568
AV-block	1.1 (0.7–1.6)	0.778	1.6 (0.6–3.9)	0.346
Echocardiographic data				
LVEF	0.9 (0.8–1.0)	0.077	1.0 (0.7–1.3)	0.824
LV mass index	1.5 (1.4–1.7)	< 0.001	1.9 (1.4–2.5)	< 0.001
LVEDV	1.7 (1.5–1.9)	< 0.001	2.0 (1.3–2.9)	< 0.001
LV lateral eʹ	0.3 (0.2–0.3)	< 0.001	0.5 (0.3–0.7)	0.001
LV septal eʹ	0.3 (0.2–0.3)	< 0.001	0.3 (0.2–0.5)	< 0.001
E/eʹ mean ratio	5.3 (4.6–6.2)	< 0.001	2.4 (1.9–3.1)	< 0.001
E/A ratio	0.9 (0.8–1.1)	0.318	1.1 (0.8–1.5)	0.525
TR Vmax	1.0 (0.9–1.1)	0.913	1.2 (0.8–1.8)	0.429
TAPSE	1.0 (0.9–1.1)	0.833	0.9 (0.6–1.3)	0.636
RV sʹ	0.9 (0.8–1.0)	0.251	0.9 (0.7–1.2)	0.648
LASV	2.2 (2.0–2.5)	< 0.001	2.1 (1.6–2.7)	< 0.001

Adjustment was performed for BMI, hypertension, diabetes, coronary artery disease (CAD), and smoking. Abbreviations as in Tables 1, 2

** ISCED (International Standard classification of education) classification 1 and 2

*** Scores of the EQ-5D (European Quality of Life 5 Dimensions 3 Level Version) and SF-8 (Short Form 8 Health Survey)

However, comorbidities and demographics that were not related to ALVDD but significantly to DDwHFpEF comprised AF with an OR of 2.6 (95% CI 1.1–5.9, *p *= 0.024), CAD with an OR of 7.2 (95% CI 3.6–14.2, *p *< 0.001), COPD with an OR of 3.9 (95% CI 1.8–8, *p *< 0.001) and female sex with an OR of 2.5 (95% CI 1.3–4.9, *p *= 0.006). Individuals with DDwHFpEF demonstrated impaired physical abilities, evidenced by the EQ5D score. Functionally, DDwHFpEF in contrast to ALVDD was associated with a prolonged QRS complex (OR 1.4, 95% CI 1.1–1.7, *p *= 0.005).

**Fig. 3:**
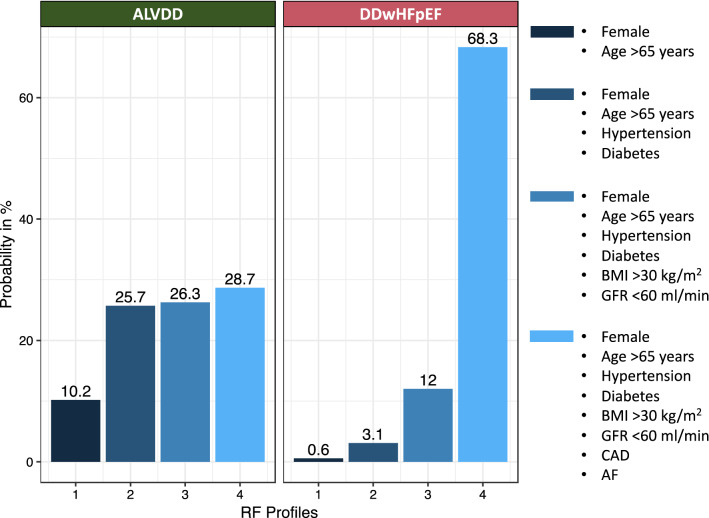
Probability of ALVDD and DDwHFpEF based on specific risk factor profiles. The probability for ALVDD and DDwHFpEF was calculated by logistic regression analysis based on the following risk factor profiles: (1) female sex, age > 65 years, no arterial hypertension, no diabetes, BMI ≤ 30 kg/m^2^, GFR ≥ 60 ml/min; (2) As 1 plus arterial hypertension, diabetes; (3) As 2 plus BMI > 30 kg/m^2^, GFR < 60 ml/min; (4) As 3 plus CAD, AF. *AF* atrial fibrillation, *ALVDD* asymptomatic left ventricular diastolic dysfunction, *BMI* body mass index, *CAD* coronary artery disease, *GFR* glomerular filtration rate, *RF* risk factor

In binary logistic regression analysis, clustering of specific risk factors increased the probability of ALVDD and DDwHFpEF (Fig. 3). While the combination of female sex, age > 65 years, diabetes, and arterial hypertension predicted a 25.7% probability for being classified in the ALVDD group, the same risk factor combination predicted DDwHFpEF by 3.1%. Adding BMI > 30 kg/m^2^, GFR < 60 ml/min, CAD, and AF to the risk factor profiles resulted in a 68.3% probability for DDwHFpEF compared to 28.7% for ALVDD.

## Discussion

The present study provides new data on the prevalence and multiple correlates of DD, both with symptomatic HF (DDwHFpEF) and asymptomatic (ALVDD), from a large sample of the first 10,000 participants of the population-based HCHS. The prevalence of DD was 12.7%. The majority of subjects with DD was asymptomatic (11.5%), while 1.3% showed overt HFpEF.

We found different patterns of comorbid conditions, risk factors, and functional parameters to be associated with either ALVDD or DDwHFpEF. After adjusting for several potential confounders, ALVDD was, among others, associated with HbA1c and arterial hypertension, whereas DDwHFpEF correlated with CAD, AF, COPD, female sex, and the width of the QRS complex.

Thereby, we identified potential research targets for further understanding the formation of both ALVDD and DDwHFpEF.

### Prevalence of diastolic dysfunction and DDwHFpEF

DD is a complex syndrome originating from the pathological interplay of left ventricular pressure decline during diastole, volume load conditions, and chamber stiffness. The assessment of DD can be performed invasively with specific high-fidelity catheters, which remains the gold-standard, or non-invasively, by the use of echocardiography [3]. When assessed non-invasively, there is no single variable that reliably reflects DD, but a combination of Doppler and native measurements is applied. Until now, numerous approaches have been proposed for reliably diagnosing and classifying DD. It is pivotal to emphasise the contrasting DD definitions and the heterogeneity both in the acquisition techniques and study populations for understanding the striking differences in the reported prevalence of DD [15]. Before the introduction of the 2016 ASE/EACVI recommendations, the reported prevalence of DD ranged between 11.1% and 36% within the normal population [16, 17]. Applying the 2016 ASE/EACVI recommendations for the diagnosis of DD, the prevalence reported seemed to be significantly reduced. Two studies by Huttin et al. and Almeida et al. showed a prevalence of 1.3% and 1.4% [7, 18]. In line with this striking antagonism, in our population-based cohort, we reported, that DD was present in 12.7% and that ALVDD was present in 11.5% of subjects aged 46 and 78 while only 0.46% of all subjects were classified in the DD group applying the 2016 ASE/EACVI recommendations (Supplements). The main novelty of the 2016 ASE/EACVI recommendations compared to previous DD algorithms was the integration of TR peak velocity and a higher E/eʹ cut off, resulting in a high specificity on the cost of a rather low sensitivity highlighted by several simultaneous echocardiographic-catheterization studies [19]. Furthermore, the ASE/EACVI recommendations classified anyone with underlying myocardial disease as DD. However, the recommendations lack a definition of relevant myocardial disease. Accordingly, in line with the studies cited above, we strictly applied the echocardiographic criteria for defining DD [7, 18]. This of course led to a lower prevalence of DD in our study than expected.

Nevertheless, our study primarily screened for diastolic dysfunction in the general population as a possible biomarker in mainly asymptomatic and only a small proportion of symptomatic subjects. For this approach, in line with most population-based studies, we chose a DD definition with a reasonable sensitivity based on the most robust, feasible, and prognostically-relevant parameters e’ velocity, E/e’ and LAVI [20, 21].

### Correlates of ALVDD and DDwHFpEF

ALVDD is considered a precursor of manifest heart failure [8]. Mechanisms underlying the formation of ALVDD are poorly understood. Despite the absence of symptoms, subjects with ALVDD demonstrated signs of cardiac remodelling including left ventricular hypertrophy and left atrial enlargement in our study, both established prognostic markers for adverse heart failure events [22].

Moreover, our results support the hypothesis, that not only the formation of HFpEF but also of ALVDD might be comorbidity-driven [23]. Consistently, in our study ALVDD and DDwHFpEF shared independently associated risk factors including age and BMI, which have been linked to the progression from ALVDD to DDwHFpEF before [8, 24]. The observed distribution of BMI in our study population is consistent with that of the Gutenberg Health Study, a German cohort study of similar design and age [25, 26]. Interestingly, the risk factors arterial hypertension and the biomarker HbA1c were associated with ALVDD but not with DDwHFpEF beyond the risk factors adjusted for. Possibly, arterial hypertension and elevated blood sugar levels might play crucial roles as driving factors especially for the early formation of DD [27]. In contrast, the widespread diseases AF, CAD, and COPD were strongly associated with DDwHFpEF but not with ALVDD and the presence of AF and CAD boosted the probability of HFpEF in our risk profile-based analysis. Population-based studies from Olmsted County, as well as results from the Framingham Heart Study, revealed, that new-onset heart failure in subjects with preserved ejection fraction was associated with AF, COPD, and CAD. [4, 28] Accordingly, the effects of AF, COPD, and CAD on a possible progression from ALVDD to HFpEF should be prospectively investigated in future studies. Additionally, the width of the QRS complex was associated with HFpEF but not with ALVDD. Accordingly, QRS duration could be either considered as a marker of heart failure or possibly represent dyssynchrony of the ventricles which might play a role in the transition from ALVDD to HFpEF. [29]

### Limitations

Our study cohort of the first 10,000 HCHS participants originates from the population of Hamburg. Hence, most study participants were of Caucasian ascend. The functional translation of our results into other populations is, therefore, limited. Therefore, our findings should be examined in other ethnic and racial groups.

Our classification of subjects as DD with and without HFpEF is based on diagnostic algorithms. There was no specific gold-standard for diagnosing DD and HFpEF, e.g., by invasive catheterization. Thus, our study did not assess the diagnostic accuracy of the different algorithms. Furthermore, dyspnoea, as the leading symptom of HFpEF, was assessed without clinical testing by a validated questionnaire.

Finally, our study setting is cross-sectional. We cannot tell which variables were the cause or the effect of ALVVD and HFpEF. Having shown multiple new associated factors of ALVDD and DDwHFpEF, further prospective studies are needed to evaluate their role in the genesis or preservation of heart failure.

### Conclusions

Our study provides new data on the prevalence and correlates of DD both with and without symptomatic heart failure in the general population. The prevalence of DD was 12.7%. Of those 11.5% were free of symptoms (ALVDD) and 1.3% suffered from HFpEF (DDwHFpEF). We identified different patterns of cardiovascular risk factors, comorbidities, and functional parameters associated with either ALVDD or DDwHFpEF. These results warrant further research concerning the exact role of the identified correlates for the formation of DD and HFpEF.

## Supplementary Information

Below is the link to the electronic supplementary material.

Supplementary file1 (DOCX 14 KB)

## Data Availability

The data underlying this article cannot be shared publicly due to the privacy of individuals that participated in the study. The data will be shared on reasonable request to the corresponding author.

## Abbreviations

AF: Atrial fibrillation
ALVDD: Asymptomatic left ventricular diastolic dysfunction
ARB: Angiotensin receptor blocker
ASE: American Society of Echocardiography
CCA: Common carotid artery
CAD: Coronary artery disease
CFA: Common femoral artery
COPD: Chronic obstructive pulmonary dysfunction
CRT: Cardiac resynchronization therapy
CVI: Chronic venous insufficiency
DD: Diastolic dysfunction
DDwHFpEF: Diastolic dysfunction with heart failure with preserved ejection fraction
GFR: Glomerular filtration rate
EACVI: European Association of Cardiovascular Imaging
ESC: European Society of Cardiology
HCHS: Hamburg City Health Study
HF: Heart failure
HFpEF: Heart failure with preserved ejection fraction
ICA: Internal carotid artery
IMT: Intima-media-thickness
LAP: Left atrial pressure
LAVI: Left atrial systolic volume indexed to body surface area
LVEF: Left ventricular ejection fraction
LVMI: Left ventricular mass index
PAD: Peripheral artery disease
QoL: Quality of life
SES: Socioeconomic status
TR: Tricuspid regurgitation
