# Supplementary material for: Diastolic dysfunction in individuals with and without heart failure with preserved ejection fraction
Source: Clin Res Cardiol. 2021 Jul 16;111(4):416–27. doi: 10.1007/s00392-021-01907-x (PMC8971165; doi:10.1007/s00392-021-01907-x)
Supplement: Supplementary file 1 — Supplementary file1 (DOCX 14 KB) [file 392_2021_1907_MOESM1_ESM.docx]

**Supplementary Files**

**Definition of Diastolic Dysfunction – 2016 recommendations**

Diastolic function was additionally assessed according to the 2016 recommendations from the American Society of Echocardiography and the European Association of Cardiovascular Imaging.[3] Subjects with LVEF≥50% were classified as normal diastolic function, indeterminate or DD on the basis of average E/e’ ratio > 14, septal e’< 7 cm/s or lateral e’<10 cm/s, tricuspid regurgitation (TR) velocity > 2.8 m/s and LAVI >34 ml/m^2^. In case of missing data for average E/e’ ratio, septal E/e’ ratio >15 or lateral E/e’ ratio > 13 were used. If more than half of the four parameters were positive, DD was assumed. If less than 50% of the criteria were positive, DD was ruled out and if two of the four parameters were positive, diastolic function was classified as indeterminate.
